# Nmnat1-Rbp7 Is a Conserved Fusion-Protein That Combines NAD+ Catalysis of Nmnat1 with Subcellular Localization of Rbp7

**DOI:** 10.1371/journal.pone.0143825

**Published:** 2015-11-30

**Authors:** Hao Chen, Darwin Babino, Stefan A. Schoenbichler, Valeryia Arkhipova, Sonja Töchterle, Fabian Martin, Christian W. Huck, Johannes von Lintig, Dirk Meyer

**Affiliations:** 1 Institute of Molecular Biology/CMBI, University of Innsbruck, Technikerstrasse 25, 6020, Innsbruck, Austria; 2 Institute of Analytical Chemistry and Radiochemistry/ CCB–Center for Chemistry and Biomedicine, University of Innsbruck, Innrain 80–82, 6020, Innsbruck, Austria; 3 School of Medicine, Department of Pharmacology, Case Western Reserve University, 2109 Adelbert Road, Cleveland, Ohio, 44106, United States of America; University Zürich, SWITZERLAND

## Abstract

Retinol binding proteins (Rbps) are known as carriers for transport and targeting of retinoids to their metabolizing enzymes. Rbps are also reported to function in regulating the homeostatic balance of retinoid metabolism, as their level of retinoid occupancy impacts the activities of retinoid metabolizing enzymes. Here we used zebrafish as a model to study *rbp7a* function and regulation. We find that early embryonic *rbp7a* expression is negatively regulated by the Nodal/FoxH1-signaling pathway and we show that Nodal/FoxH1 activity has the opposite effect on *aldh1a2*, which encodes the major enzyme for early embryonic retinoic acid production. The data are consistent with a Nodal-dependent coordination of the allocation of retinoid precursors to processing enzymes with the catalysis of retinoic acid formation. Further, we describe a novel *nmnat1-rbp7* transcript encoding a fusion of Rbp7 and the NAD^+^ (*Nicotinamide adenine dinucleotide*) synthesizing enzyme Nmnat1. We show that *nmnat1-rbp7* is conserved in fish, mouse and chicken, and that in zebrafish regulation of *nmnat1-rbp7a* is distinct from that of *rbp7a* and *nmnat1*. Injection experiments in zebrafish further revealed that Nmnat1-Rbp7a and Nmnat1 have similar NAD^+^ catalyzing activities but a different subcellular localization. HPLC measurements and protein localization analysis highlight Nmnat1-Rbp7a as the only known cytoplasmic and presumably endoplasmic reticulum (ER) specific NAD^+^ catalyzing enzyme. These studies, taken together with previously documented NAD^+^ dependent interaction of RBPs with ER-associated enzymes of retinal catalysis, implicate functions of this newly described NMNAT1-Rbp7 fusion protein in retinol oxidation.

## Introduction

Retinoids (vitamin A and its derivatives) are important for various physiological functions in adult vertebrates and are also critical for embryonic development. Studies on the developmental functions of retinoids have mainly focused on the vitamin A derivative retinoic acid (RA). Analyses in vertebrate model organisms such as zebrafish, frog, chicken, and mouse [1] uncovered conserved embryonic requirements in axis formation, neural differentiation, hindbrain patterning, regulation of limb development, and during organogenesis of pancreas, heart, kidney and lung [2–5]. RA serves as a signaling molecule that regulates gene expression via the heterodimeric complex of the nuclear cognate ligand receptors RARs (RA receptors) and RXRs (retinoid X receptors) [6]. These are ligand-dependent transcription factors that switch from potential repressors to transcriptional activators upon binding of RA.

Biologically active retinoids are generated by enzymatic conversion of diet-derived, or in the case of the embryonic tissue, maternally-derived precursor molecules. Studies over the past decades have revealed complex molecular mechanisms for retinoid uptake, metabolism, transportation and storage [7]. RA synthesis requires function of several NAD^+^/NADP^+^-dependent enzymes such as aldehyde-dehydrogenases (ALDHs) for the oxidation of retinaldehyde to RA, short-chain dehydrogenase/reductases (SDRs) including retinol dehydrogenases (RDHs), and alcohol dehydrogenases (ADHs) for conversion of retinol to retinaldehyde [8–11]. Retinoids are chemically reactive and hydrophobic molecules, therefore, animals have evolved specific proteins, namely retinol binding protein (RBP), cellular retinoic acid-binding proteins (CRABPs) and cellular retinaldehyde-binding protein (CRALBPs) for protecting and transporting retinoids in the aqueous compartments of the body [8]. RBPs are members of the intracellular lipid binding proteins that display a high binding affinity to all-*trans*-retinol (ROL) [12–14]. In vertebrates different types of RBP protein facilitate intracellular- (Rbp1, Rbp2, Rbp5, Rbp7), inter-photoreceptor- (Rbp3/ IRBP) and plasma-specific transport (Rbp4) mainly of ROL [15]. Increasing evidence implicates intracellular RBPs not only in ROL transport, but also as having a central role in balancing retinoid activity and metabolism. Rbp1 (formally referred to as cRbp1) was found to directly bind to microsome associated LRAT and to modulate the LRAT catalyzed conversion of retinol to retinyl esters [8, 16]. Rbp1 also showed an NAD^+^ dependent interaction with microsomal SDRs and activities in retinol oxidation to retinal for retinoic acid synthesis [8, 16]. Activities in retinoid homeostasis were also described for Rpb7 (cRbpIII in mouse, and cRbpIV in humans). While mouse *Rbp7* mutants were viable, the mammary gland and the milk from lactating mutant females displayed abnormal retinoid levels, in particular decreased retinyl ester levels [17]. The data suggest a more general role of intracellular Rbps in facilitating synthesis of retinyl ester, and in particular of retinyl palmitate, from retinol [18].

To our knowledge, studies about *rbp* genes in non-mammalian systems have been mainly conducted in zebrafish [19]. However, functional studies on zebrafish *rbp* genes until recently were limited to zebrafis*h rbp4* [20]. The knockdown of *rbp4* by injected antisense morpholinos revealed a role of *rbp4* in the formation of the yolk extension and liver bud and for the migration of liver progenitor cells at later embryonic stages. The various intracellular *rbp* genes show diverse expression patterns that suggest tissue specific activities. Earlier studies revealed locally restricted expression of *rbp1* (originally termed *rbp1b*) in the developing gall bladder, of *rbp2a* (originally termed *rbp2*) in yolk syncytial layer (YSL), intestine and retina, of *rbp2b* in YSL and liver, and a more complex and dynamic expression for *rbp5* (originally termed *rbp1*) in nervous system, intestine and YSL [21, 22]. More recently, two *rbp7* orthologs with distinct embryonic expression mainly in the YSL and forerunner cells (*rbp7a*) and in somites (*rbp7b*) have been described [23].

In this study we focus on the early embryonic role of the *rbp7a* gene, which very recently was found to interact with Nodal/TGF-beta signaling pathway during left-right patterning [24]. In particular, morpholino knock-down of *rbp7a* was shown to cause reversed heart looping in otherwise normal appearing embryos. The data suggest a local role of *rbp7a* in forerunner cells that is required for the establishment of a left side-specific Nodal activity domain in the embryo. We now find that *rbp7a* is negatively regulated by Nodal signaling via the transcription factor FoxH1. As Nodal and FoxH1 are also a key activators of the RA producing enzyme Aldh1A2 our data suggest a complex interplay between the Nodal and RA signaling pathways. Further, we describe a novel conserved *rbp7* mRNA isoform encoding a fusion protein of Rbp7 and nicotinamide mononucleotide adenylyltransferase 1 (Nmnat1), a key enzyme for the synthesis of NAD^+^. We show that Nmnat and the Nmnat1-Rbp7a fusion protein possess similar activities in NAD^+^ catalysis but a different subcellular localization. As NAD^+^ is an essential co-factor for several enzymes of retinoid catalysis (ADHs, RDH and ALDHs) and has impact on the molecular interactions of Rbps, the data suggest Nmnat1-Rbp7a functions in linking ROL transport and retinoid metabolism.

## Material and Methods

### Zebrafish lines

AB wild type embryos, *foxH1*/MZ*sur* ^*m768/m768*^ mutants [25] and embryos deficient for the Nodal co-receptor oep (MZ*oep* ^*m134/m134*^) [26] were raised and staged as described [27]. Embryos were kept in E3 solution at 28.5°C with or without 0.003% 1-phenyl-2-thiourea (Sigma) to suppress pigmentation and staged according to somite number or hours post-fertilization (hpf). This study was approved by the Austrian Bundesministerium für Wissenschaft, Forschung und Wirtschaft (BMWFW, GZ BMWF-5-032/0002-C/GT/2007 BMWF-66.008/0018-II/3b/2013), and all procedures were carried out in accordance with the approved guidelines.

### cDNA isolation and gene cloning

Reverse Transcriptase PCR on 12 somite stage zebrafish mRNA was used to amplify full-length cDNAs of *rbp7a*, *nmnat1* and *nmnat1-rbp7a* including parts of the 5’ UTR sequence (for primer sequences see S1 Table). The PCR products were cloned into pGEMT-easy vector (Promega) and sequenced. For mRNA production the inserts were cut with EcoRI or BamH1-XholI fragment and sub-cloned into corresponding sites of the pCS2+ vector. For the generation of eGFP fusion constructs the coding sequences were end-modified by PCR amplification to fit into BamHI-XbaI sites of a pCS2+-C-terminal eGFP plasmid.

### RNA isolation and cDNA synthesis

Total RNAs were prepared by using the Trizol (Invitrogen) according to the manufacturer’s instructions. All RNA was digested by RNase-free DNase I. About 3 mg RNA was used as template for reverse transcription using Superscript II kit (Invitrogen).

### Whole mount in situ hybridization (WISH) analysis

Whole mount *in situ* analyses were performed as described [28]. The corresponding digoxigenin-UTP labeled antisense probes for *rbp7a* and *aldh1A2* were generated from linearized plasmid DNA by in vitro translation using digoxigenin-UTP-Mix (Roche Applied Science) and T7 or SP6 RNA polymerase (Fermentas).

### RT-PCR and quantitative PCR (RT-qPCR)

Semiquantitative reverse transcription PCR (RT-PCR) was performed using Dream Taq mix (Fermentas). *β-actin* was used as the control for normalizing cDNA amounts. Competition PCR for analyzing relative expression levels of *rbp*7a, *nmnat1* and *nmna1-rbp7a* was performed by adding three primers instead of two. Quantitative Real-time PCR (qPCR) reactions were performed in a Biorad CFX Connect using SYBR Green Mix (ABI) or Eva Green Mix (Bio-Rad). The PCR running protocol was based on the ABI products introductions. *β*-actin and *ef1α* were used as controls to normalize different samples. The data analysis was performed as described [29, 30]. For primer information see S1 Table.

### Electrophoretic Mobility Shift Assays (EMSA)

EMSA tests were performed as described [31] using *in vitro* synthesized (TNT Quick-coupled Transcription/Translation kit, Promega). The following oligonucleotides containing putative FoxH1 binding sites were used: S1: CTGAATACACAAG; S2: CCTTGTGTATTTGC; S3: TGGTGTGTATTTA; S4: TTTAATACACAACA. Oligos were end-labeled using γ-^32^P-Adenosine 5’-triphosphate (PerkinElmer) and polynucleotide kinase (Fermentas, EK0031). The binding reaction was performed using 25,000 cpm for each oligonucleotide, 2 μl protein in the presence of 0.1 mg/ml of poly(dI-dC) and 1 × binding buffer (5 × buffer: 60 mM HEPES pH 7.9, 20 mM Tris—HCl pH 7.9, 300 mM KCl, 60% (v/v) glycerol, 5 mM DTT and 5 mM EDTA). The mix was incubated for 45 min at room temperature. For competition assays, FoxH1 protein was 15 min pre-incubated with 100 fold higher concentration of unlabeled competitor oligos before adding the labeled probes. Binding was analyzed by running a native 5% polyacrylamide gel, followed by overnight exposure of film (GE Healthcare, Amersham Hyperfilm^™^ MP) at −80°C before development.

### Chromatin Immunoprecipitation Assay (ChIP)

Anti-eGFP antibody (Torrey Pine Biolabs, 10μg/1000 embryos) was used to precipitate eGFP-tagged FoxH1 protein from embryos that had been injected with *eGFP-foxH1* mRNA at the 1–2 cell stage. To exclude occupation of FoxH1 binding-sites by endogenous FoxH1 proteins, *eGFP-foxH1* was injected into MZ*sur* ^*m768*^ embryos in which a point mutation within the forkhead domain abolished DNA-binding of FoxH1 [25]. ChIP experiments were performed as described [32] with some minor changes. In total, about 4000 *foxH1* mutant embryos (MZ*sur* ^*m768/m768*^) were injected each at 1–2 cell stage with 6ng/μl (~30pg/embryo) of *eGFP-foxH1* mRNA, encoding full length FoxH1 with an N-terminal eGFP tag. Embryos were collected and cross-linked at 6hpf. The antibody was directly added to the cross-linked samples for overnight at 4°C instead of incubating it with magnet beads (Invitrogen) before adding it to the sample. The next day, the beads were added to IP down protein-DNA complexes.

### Microinjections of morpholinos and mRNAs

The following morpholinos (Gene Tools) were used: MO-ATG targeting the translating start of the *rbp7a m*RNA (5’-ATGTTCCACAGAAGCTCACAGGCAT-3’; the ATG complementary sequence is underlined); MO*-Δex2* was targeting the intron1-exon2 boundary of the *rbp7a* gene (5'-gatccCTGTGACAAGACAAAGTTTA-3', lower caps indicate first intron sequences); *p53-*MO: GCGCCATTGCTTTGCAAGAATTG (standard from Gene Tools). Sense capped mRNAs were synthesized by using SP6/T7 mMESSAGE mMACHINE for *in vitro* transcription (Ambion). Indicated amounts of MO and mRNA were injected at 1–2 cell stage.

### Extraction of Retinoids and NAD^+^ from embryos for the HPLC measurements

Extractions and HPLC measurements of retinoids were based on previously established protocols [33]. For the determination of apolar retinoids, 200μl of 2 M NH_2_OH and 200 μl of 100% MeOH were added to the embryos and homogenized for 2 minutes. After 10 minutes of incubation at room temperature, 800 μl of acetone were added and retinoids were extracted twice in 500 μl of hexane. The collected supernatants were vacuum dried and dried pellets re-suspended in HPLC solvent. Retinoid analyses were performed with a normal phase Zorbax SIL (5μm, 4.6 × 150 mm) column (Agilent Technologies, Santa Clara, CA). For retinyl ester separation, a linear gradient of 0.5% ethyl acetate in hexane over 15 minutes followed by 20 minutes of 10% ethyl acetate in hexane was used with a continuous flow rate of 1.4 ml/min with detection at 325 nm. For determination of RA levels, embryos were collected and homogenized in 350 μl of PBS buffer (137 mm NaCl, 2.7 mm KCl, 7.3 mm Na_2_HPO_4_, 1.47 KH_2_PO_4_, pH 7.2) and 150 μl of ethanol. Then 750 μl of ethylacetate/methylacetate (8:1, v,v) were added. The supernatant was collected and transferred to a new reaction tube. Extraction of the aqueous phase was repeated and the collected supernatants were vacuum dried and pellets re-suspended in HPLC solvent. The HPLC solvent was *n*-hexane and ethylacetate (81:19, v,v) containing 12.5 μl of acetic acid/100 ml. Retinoid analyses were performed with a normal phase Zorbax SIL (5μm, 4.6 × 150 mm) column (Agilent Technologies, Santa Clara, CA). For NAD^+^ analysis, NAD^+^ was extracted according to the method of Wang et al [34]. For HPLC measurements were performed on a Shimadzu 10A system coupled to a Shimadzu SPD-M10A_VP_ diode array detector (Shimadzu, Kyoto, Japan). Solvent A was an aqueous 0.05 M KH_2_PO_4_/K_2_HPO_4_ buffer pH 7 and solvent B was acetonitrile. An isocratic flow with 3% B and 1 ml/min was performed for 6 min. NAD^+^ was detected at 259 nm. Injection volume was 40 μl and the column oven was set to 25°C. The stationary phase was a Thermo Scientific BDS Hypersil C18 column (Thermo Fisher Scientific, Waltham, USA), 250 x 4 mm with a particle size of 5 μm. For each NAD^+^ or retinoid extraction experiment 100–200 embryos were collected at 6hpf or 14hpf, respectively.

### Cell culture and cell transfection

Human embryonic kidney (HEK) 293 cells were cultured in Dulbecco’s Modified Eagle’s Medium with glucose, L-glutamine, 10% fetal calf serum, penicillin and streptomycin (Lonza) in a humidified 5% CO_2_ atmosphere at 37°C. The day before transfection, HEK293 cell (0.4x10^6^ cells) were seeded in 6 well-plates. Transfection was performed according to the manufacturers instructions using 2.5μg plasmid DNA and 9μl Lipofectamine2000 Reagent (Invitrogen) per well. Cells were documented 24–30 hours after transfection.

### Western-Blot analyses

Western-blot-analyses were performed with normalized protein amounts by using anti-GFP (1:5.000; Torry Bines Biolabs, No.:TP401), HRP-conjugated secondary Anti-rabbit (1: 10.000; Jackson Immuno Research Lab. No.; 111-035-045) and ECL detection (Amersham No.: RPN2235). Images were documented on a ChemiDoc™ XRS+ System (BioRad). ImageJ was used for data quantification.

Protein samples were generated form 6hpf embryos. Embryos were manually dechorionated and pools of 20 embryos were stored in liquid nitrogen. Whole-embryo extracts was generated by directly dissolving 10 embryos in 50 μl RIPA buffer.

For microsomal preparation frozen embryos were suspended in 0,4 ml ice cold M-buffer (10μM Tris pH 8; 1mM PMSF; 1x protease inhibitor mix from GE-healthcare). After 15 minutes of incubation on ice, samples were passed several times through 0,4mm syringe needles. Insoluble components including the nuclei were pelleted by 20 minutes centrifugation (750g, 4°C, termed ‘nuclear’). The supernatant was transferred to ultracentrifuge tubes and microsomes were pelleted by 20 minutes ultracentrifugation (200.000g, 4°C). Pellets from both centrifugation steps (1: ‘nuclear extract; 2: microsome extract) were resuspended in 40μl M-buffer and stored at -80°C. Aliquots of 2μl were used to determine protein content using photometry and Coomassie blue-stained polyacrylamide gels. Approximately 15μl of microsome extract (~1,7 embryos), 3 μl of the nuclear extract (~1,5 embryos) dissolved in 12μl RIPA buffer, and 10μl whole embryo extract (~7,5 embryos) were used.

### Statistical analysis

All values of triple biological repeats were calculated into means and ±S.D. Graphs were produced using Prism 6 and Excel. Unpaired T-test or T-test with Welch’s correction was used for all comparison analyses by SPSS 20 or Prism 6. In all analyses, a P-value less than 0.05 was considered to be statistically significant (*P<0.05; **P<0.01; ***P<0.001).

## Results

### Complementary regulation of *rbp7a* and *aldh1A2* by Nodal signaling

**Fig 1:**
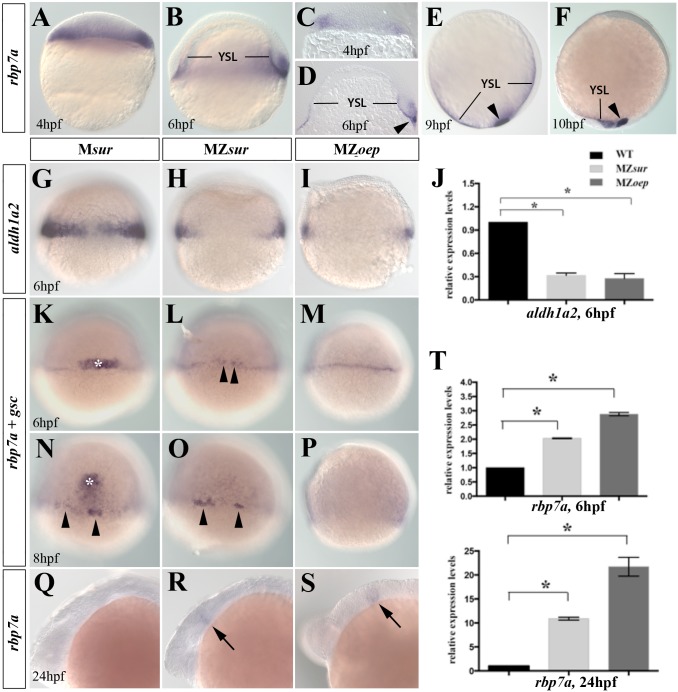
Complementary regulation of *rbp7a* and *aldh1a2* by Nodal signaling. [A-F] Lateral views of whole mount *in situ* stains of *rbp7a* in wild type embryos at 4hpf [A, C] 6hpf [B,D], 9hpf [E] and 10hpf [F]; [C-D] vibratome sections of 4hpf and 6hpf embryos. *rbp7a* signals are found in marginal cell [A-D], YSL, and in forerunner cells (arrowhead in [D-F]). [G-J] Marginal expression of *aldh1a2* is strongly reduced in 6hpf MZ*sur* [H] and MZ*oep* [I] embryos; [J] RT-qPCR verified reduced expression levels of *aldh1a2* in MZ*sur*, MZ*oep* as compared to wild type embryos. [K-P] Whole mount *in situ* hybridization for *rbp7a* (arrow heads) and *goosecoid* (*gsc*) (white asterisks) performed at 6 and 8 hpf in M*sur* ^*+/-*^ as control [K, N], MZ*sur* [L, O] and MZ*oep* [M, P] embryos. Note that expression levels of *rbp7a* around the YSL were similar among the *gsc* positive embryos and the *gsc* negative MZ*sur* [B, E] and MZ*oep* [C, F] mutants. [Q-S] *rbp7a* expression at 24hpf. Arrows mark *rbp7a* signals in the posterior midbrain that were present in MZ*sur* [R] and MZ*oep* embryos [S] but not in control embryo [Q]. [T] RT-qPCR results for *rbp7a* in of 6hpf and 24hpf embryos. Graphed is the mean and SEM from triplicate experiments. Error bars indicated the SEM. Unpaired T-test was used to test the significance (*P<0.05).

The Nodal and RA signaling pathways are two key regulators of embryonic patterning. While bidirectional interactions between these pathways are well documented, the molecular details have not been fully characterized. Recently, rbp7a has been suggested to contribute to the RA/Nodal interaction network [24]. Consistent with this notion, we had previously identified *rbp7a* in screen for Nodal regulated genes [35]. Detailed analyses of embryonic *rbp7a* expression revealed the first specific *rpb7a* signals in marginal cells of the late blastula and early gastrula embryo (Fig 1A–1D). During gastrulation, expression was restricted to the YSL and forerunner cells (Fig 1B and 1D–1F). As the early marginal expression of *rbp7a* displayed similarities with that of *aldh1A2* [36], encoding the key enzyme for early embryonic retinal to RA conversion, we speculated that these genes may be similarly regulated. Previous studies in mice reported a role of Nodal signaling and in particular of the transcription factor Foxh1 in controlling activation of *aldh1A2* [37]. Consistent with a similar cross-talk between Nodal/FoxH1 and RA-signaling in zebrafish, we found that *aldh1A2* expression is strongly reduced in both Nodal-signaling deficient MZ*oep* mutants and MZ*sur/foxH1* mutants during gastrulation (Fig 1G–1J). In contrast, whole mount *in situ* hybridization analyses for *rbp7a* expression revealed a very similar pattern in control and mutant embryos at 6 and 8 hpf (Fig 1K–1P). The embryos showed weak signals in the YSL and in case of control and MZ*sur* stronger signals in forerunner cells (arrow heads, asterisk mark co-stained *gsc* signals, which was used to distinguish embryos derived form cross of a homozygous female and a heterozygous male). Corresponding forerunner signals were missing in MZ*oep* embryos as they lack these cells (Fig 1M and 1P). While *rbp7a* signals could not be detected in whole mount mRNA analyses of 24hpf wildtype embryos (Fig 1Q), MZ*sur* and MZ*oep* mutants both showed an ectopic signal in the posterior midbrain (Fig 1R and 1S). Quantitative RT-PCR analyses revealed an increase, rather than decrease, in *rbp7a* transcripts in 6hpf MZ*sur* and Mz*oep* embryos as compared to control embryos (Fig 1T). Increased expression was even more striking at 24 hpf when *rbp7a* mRNA increased more than 10 and 20 fold in MZ*sur* and MZ*oep* mutants, respectively (Fig 1T). The data demonstrate opposite effects of Nodal FoxH1-signaling on *rbp7a* and *aldh1a2* expression.

### Characterization of a potential Foxh1-regulated proximal *rbp7a* enhancer

**Fig 2:**
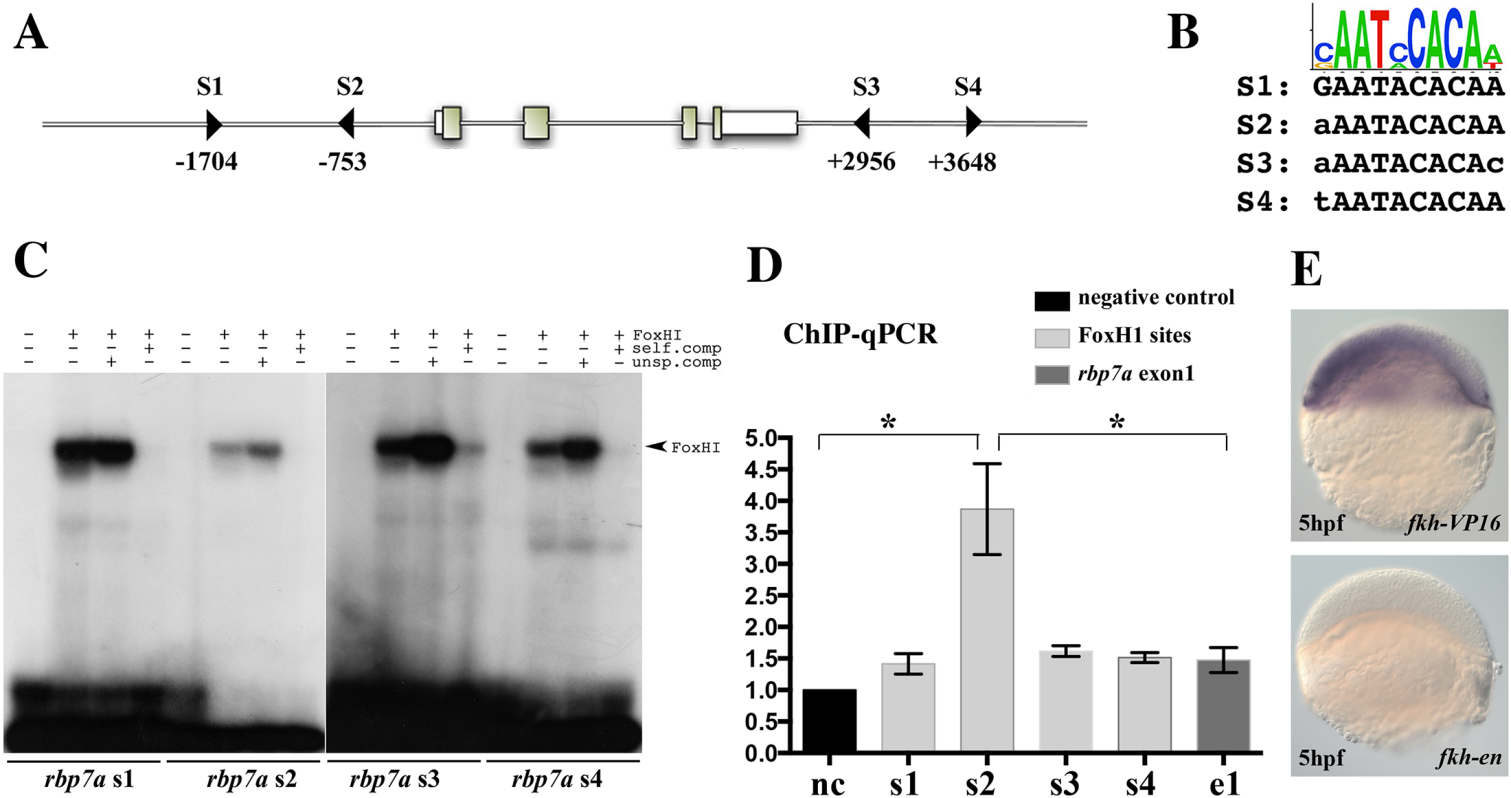
*rbp7a* is a direct target of FoxH1. [A] Schematic drawing of the *rbp7a* gene highlighting 4 potential FoxH1 binding sites (triangles: S1, S2, S3, and S4). Exons are indicated by boxes, the numbers correspond to the distance form the transcriptional start site. [B] Sequence comparison of the mouse FoxH1 consensus log [37] with the four potential sites in *rbp7a*. [C] *In vitro* EMSA studies with translated FoxH1 protein with oligonucleotides containing the four potential FoxH1 binding sites (sequences and positions were shown in [A, B]). Competition experiments with unspecific (unsp.comp) and specific (self.comp) unlabeled oligonucleotides were added to verify the binding specificity. [D] *In vivo* chromatin immunoprecipitation (ChIP-qPCR) experiments performed with 6hpf *eGFP-foxH1* mRNA injected MZ*sur* embryos. Bars show the enrichments of DNA fragments in the regions of FoxH1 binding sites in relation to a negative control region (*rhodopsin* promoter region) that was lacking FoxH1 binding sites (*P<0.05; error bars indicated the SEM). [E] Induced and depleted *rbp7a* expression in 5hpf wild type embryos after injection of *fkh-vp16* and *fkh-en* mRNA, respectively.

As FoxH1 is known to function mainly as a transcriptional activator, we investigated whether repression of *rbp7a* occurred by direct protein/DNA interaction. *In silico* screening of the *rbp7a* genomic region for the consensus motif of FoxH1 [37] revealed four putative binding sites (S1, S2, S3 and S4) (Fig 2A and 2B). Electrophoretic mobility shift assays (EMSAs) confirmed *in vitro* binding of the zebrafish FoxH1 protein to all four binding sites (Fig 2C). The specificity of these bindings was further confirmed by competition assays with DNA containing a mutated FoxH1 consensus sequence and with unlabeled oligonucleotides. To assess whether FoxH1 binds to these sites *in vivo*, we carried out chromatin immunoprecipitation (ChIP) assays with 6 hpf embryos. Analysis of the immunoprecipitated DNA fragments by quantitative PCR revealed an approximately 3.5-fold enrichment of DNA containing the S2 binding site (Fig 2D), but no enrichment for the S1, S3 or S4 binding sites. Finally, injection of mRNA encoding a transcription activating form of FoxH1 (*fkh-VP16*) or a constitutive repressing FoxH1 form (*fkh-en*) [25] caused ectopic expressions or loss of *rbp7a* expression, respectively (Fig 2E). These results are consistent with a potentially direct repression of *rbp7a* by FoxH1.

### Expression of a novel and conserved splice variant encoding an Nmnat1-Rbp7a fusion protein

**Fig 3:**
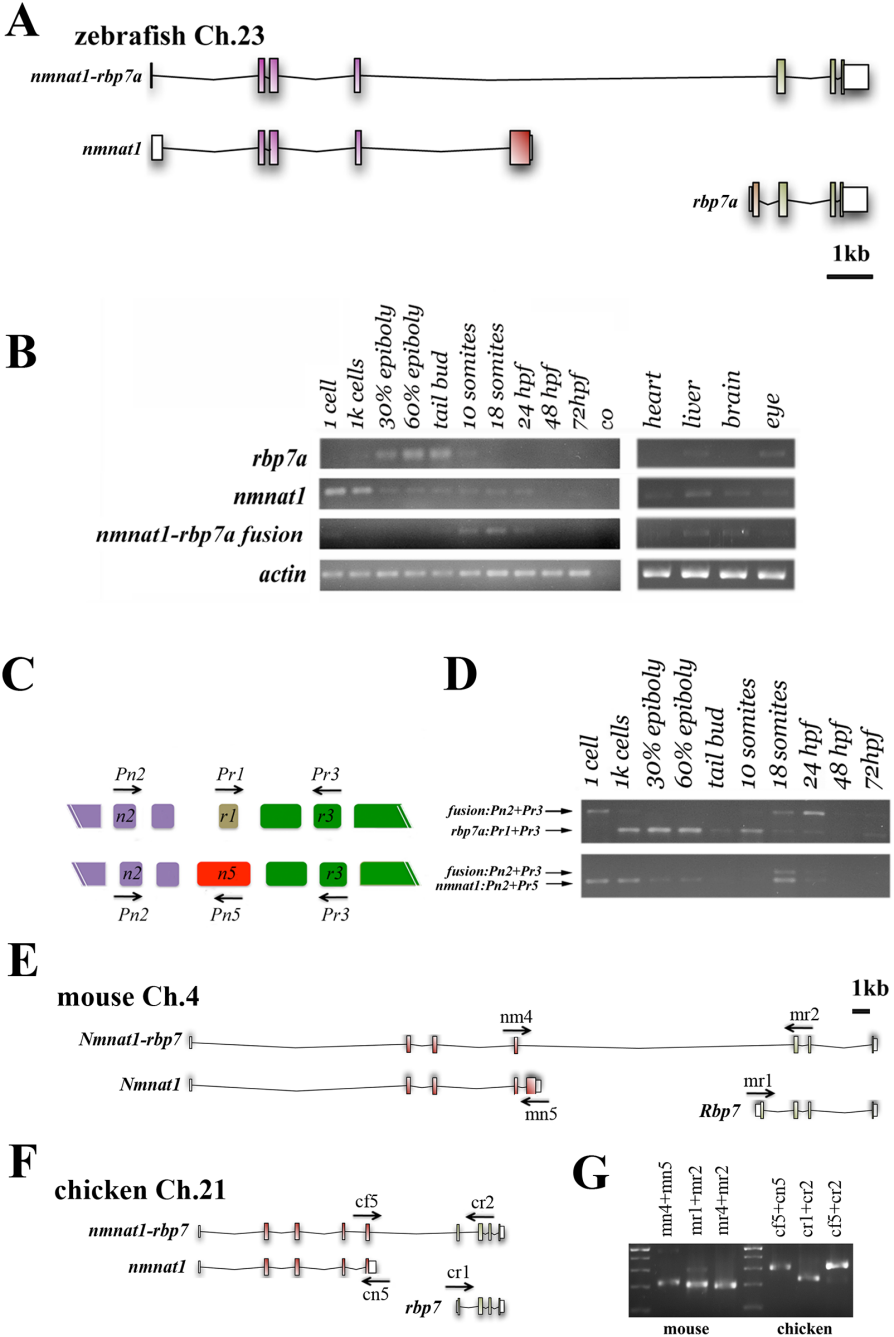
*rbp7a* forms a conserved fusion gene with *nmnat1*. [A] Schematic representation of the exon usage in *nmnat1*, *rbp7a* and *nmnat1-rbp7a* transcripts. Size of exons (boxes) and distances corresponds to the position in the genome. [B] Semi-quantitative RT-PCR analyses of *rbp7a*, *nmnat1*, and *fusion* isoform; mRNA generated from indicated embryonic stages and adult tissues. *β-actin* was used as a control for cDNA input levels. [C-D] Competitive PCRs reveal the ratios of different isoforms expression level during developmental stages. [C] Schematic indicating primers used for competitive amplification of *rbp7a* (Pr1+Pr3) versus *nmnat1-rbp7a* (Pn2+Pr3) in the upper panel and *nmnat1* (Pn2+Pn5) versus *nmnat1-rbp7a* in the lower panel. The different isoform exons are colored respectively; purple: *nmnat1*, green: *rbp7a*; the unique exon which belongs to *nmnat1* or *rbp7a* is shown in red and brown. [D] Competition RT-PCR in different embryonic stages, note the temporal changes in ratios of long (*nmnat1-rbp7a*) versus short (*rbp7a* and *nmnat1*) PCR products, which were indicated the different dominated expression time windows for isoforms. [E, F] Schematic representation of exon usage of *Rbp7*, *Nmnat1*, and the *Nmnat1-rbp7* transcriptions in mouse [E] and chicken [F]. [G] RT-PCR analyses of embryonic mRNA form mouse (E14) and chicken (d9) confirming expression of *Nmnat1*, *Rbp7* and *Nmnat1-rbp7* transcripts.

Upon analyzing the *rbp7* EST sequences, we found a unique long *rbp7a* EST entry, which is lacking the first *rbp7a* exon and instead contains 4 exons from the *5’* neighboring gene *nmnat1* (Fig 3A). To exclude that this EST represents a splice artifact, we performed semi-quantitative RT-PCR analyses with mRNA preparations isolated from different embryonic stages and adult tissues. Using a pair of primers flanking the fusion site, we detected *nmnat1-rbp7a* fusion specific PCR-products in one-cell stage embryos, segmentation stage embryos (tailbud to 24hpf), and in adult liver and brain (Fig 3B). Further analyses of the same cDNA samples also revealed that the expression of the *nmnat1-rbp7a* fusion isoform was different from that of *nmnat1* and *rbp7a* (Fig 3B). Consistent with the whole mount expression analyses, *rbp7a* RT-PCR-products were mainly detected during gastrula stages (30% epiboly to tailbud), while *nmnat1* signals were found in all stages analyzed, with the strongest signals peaking during maternally regulated stages (1 cell and 1k-stage). To confirm the different developmental expression profiles and to define the relative expression levels of *nmnat1*, *rbp7a* and *nmnat1-rbp7a*, we performed competitive PCRs for these isoforms with cDNA from different stage embryos. In these studies, the primer pairs flanking the *nmnat1-rbp7a* fusion site (Pn2 + Pr3) were combined with a third primer that localized either to the last exon of nmnat1 (Pn5) or to the first exon of *rbp7a* (Pr1) (Fig 3C and 3D). The data revealed *nmnat1-rbp7a* fusion transcripts as the prevailing *rbp7a* isoform in zygotes and between 18-somite stage and 24hpf, and they suggested that expression levels of *nmnat1-rbp7a* at late somite stages were slightly below those of *nmnat1*.

### Expression of *nmnat1-rbp7* is conserved in different vertebrate species

As a next step we asked whether occurrence of the *nmnat1-rpb7* splice variant was restricted to zebrafish. Investigation of the *rbp7* and *nmnat1* genomic regions in human, mouse, chicken, fugu (*Fugu rubripes)* and medaka (*Oryzias latipes*) showed that the genomic organization of these adjacent genes was well conserved during vertebrate evolution (not shown). RT-PCR analyses of RNA extracted from E14 mouse and 19 somite stage chicken embryos with primers designed to detect a potential *nmnat1-rbp7* fusion yielded PCR-products from cDNA preparations of both organisms (Fig 3E and 3F). Sequencing of these PCR-products confirmed amplification of an *nmnat1-rbp7* fusion isoform and revealed some species-specific differences in exon usage. In fish and mouse, the fusion was found between exon 4 of *nmnat1* and exon 2 of *rbp7*; thereby skipping *nmnat1*-exon5 and *rbp7*-exon1 (Fig 3E). In chicken, the fusion was between parts of *nmnat1*-exon 5 and *rbp7*-exon1, indicating alternative splice sites within these exons (Fig 3F). The adjacent genomic position of *nmnat* and *rbp* gene family members is not only restricted to *nmnat1/rbp7*, but also can be found for *rbp1* and *nmnat3*. Thus, we tested whether there is a similar fusion variant between these two genes. However, RT-PCR studies on embryonic mRNAs from zebrafish, mouse and chicken with primers pairs flanking potential fusion sites between *nmnat3* and *rbp1* (*rbp1b*) revealed no evidence for the existence of mRNAs that encode *nmnat3*-*rbp1b* fusion proteins (data not shown). Taken together, these analyses proved the existence of a novel conserved transcript that combines *nmnat1* and *rbp7* genes. Further, they show that zebrafish *nmnat1-rbp7a*, *nmnat1* and *rbp7a* are expressed under the control of distinct developmental and cell specific regulatory mechanisms.

### Embryonic NAD+ levels are similarly unregulated by Nmnat1 and Nmnat1-rbp7a injection

**Fig 4:**
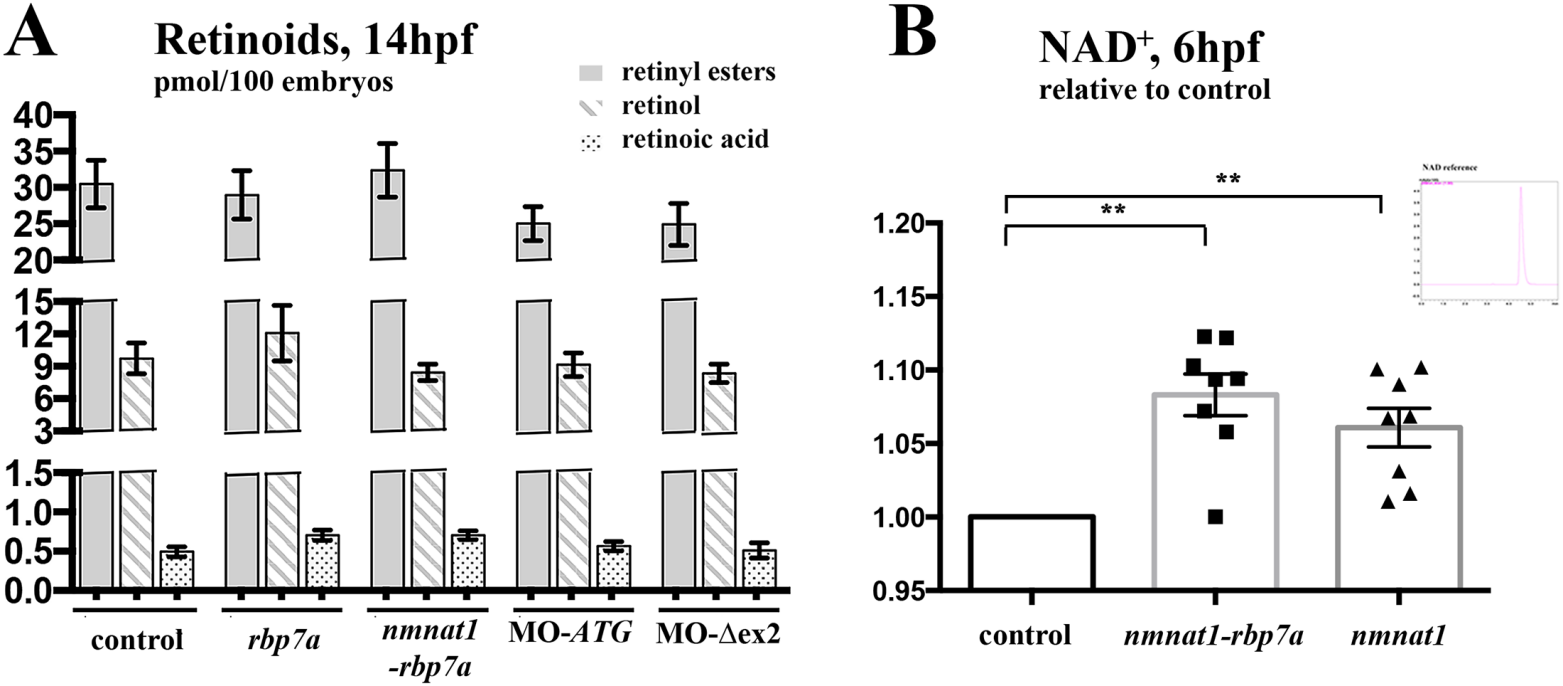
Retinoid and NAD^+^ levels in mRNA in Morpholino injected embryos. [A] HPLC measurements show no significant changes in of ROL, RE and RA levels in 14hpf embryos injected with either RNA encoding Rbp7a, Nmnat1-Rbp7, or morpholinos blocking *rbp7a* translation (MO-ATG) and proper splicing of rbp7a exon 2 (MO-Δex2). For each dataset three independent treatments were performed and 100 embryos each were collected at 14hpf from each group of mRNA injected, morphant, and non-injected embryos. [B] NAD^+^ levels increase after over-expressions of *nmnat1* and *nmnat1-rbp7a* fusion mRNA. Spectral peaks of HPLC measurements shows the peak characteristic of NAD^+^ reference. For each experiment, three times 100 embryos at 6hpf were collected in different treatment or wild type groups Error bars indicated the SEM (**P<0.01).

To assess functional properties of Rbp7a and Nmnat1-Rbp7a, different *in vivo* gene-knock down and gain-of function experiments were conducted. Morpholino injection was used to either selectively block translation of *rbp7a* transcripts (MO-ATG) or to induce skipping of the *rbp7a* and *nmnat1-rbp7a* shared *rbp7a*-exon 2 (MO-Δex2), respectively (Fig 3, see also S1 Fig and Material and Methods). Based on the role of Rbp7 in delivering retinol to Lrat, we speculated that rbp7a morphants would show similar early patterning defects to lrabt morphants. In contrast to this prediction, injection of up to 4ng/embryo of both morpholinos resulted in morphologically normal looking 20hpf embryos. While higher morpholino amounts (8ng/embryo) caused severe neuronal and mesoderm patterning defects, majority of these defects was rescued by co-injection of p53 morpholinos (not shown). This suggests that Rbp7a and Nmant1-Rbp7a are dispensable for Lratb functions that higher morpholino amounts caused p53-dependent off target effects [38]. Furthermore, HPLC measurement of all-*trans*-retinol, retinyl ester and RA levels revealed no significant changes in 14 hpf control embryos and in 14 hpf embryos injected with either *rbp7a* or *nmnat1-rbp7a* mRNA, or with *rbp7* morpholinos (Fig 4A and S2 Fig). This shows, that neither induction nor loss of Rpb7a function has a major effect on early embryonic retinoid homeostasis and RA signaling. Next we compared Nmnat1 and Nmnat1-rbp7a protein activities. Nmnat proteins convert nicotinamide mononucleotide (NMN) to NAD^+^, which serves as an electron carrier in numerous redox reactions, including the oxidation of retinol to retinal [11, 39]. Although synthesis of NMN is supposed to be the rate-limiting step in NAD^+^ synthesis [40, 41], we found that injection of either *nmnat1* or *nmnat1-rbp7a* mRNAs (100 pg/embryo) caused a weak but significant increase in NAD^+^ levels as compared to control embryos (Fig 4B and S2 Fig). In three independent sets of HPLC measurement on 60% epiboly embryos, this increase ranged from 3% to 10% and 7% to 9% for *nmnat1* and *nmnat1-rbp7a* injections, respectively, showing that both proteins have a similar capacity for NAD^+^ synthesis in the embryo.

### Different sub-cellular location of Nmnat1 and Nmnat1-Rbp7a

**Fig 5:**
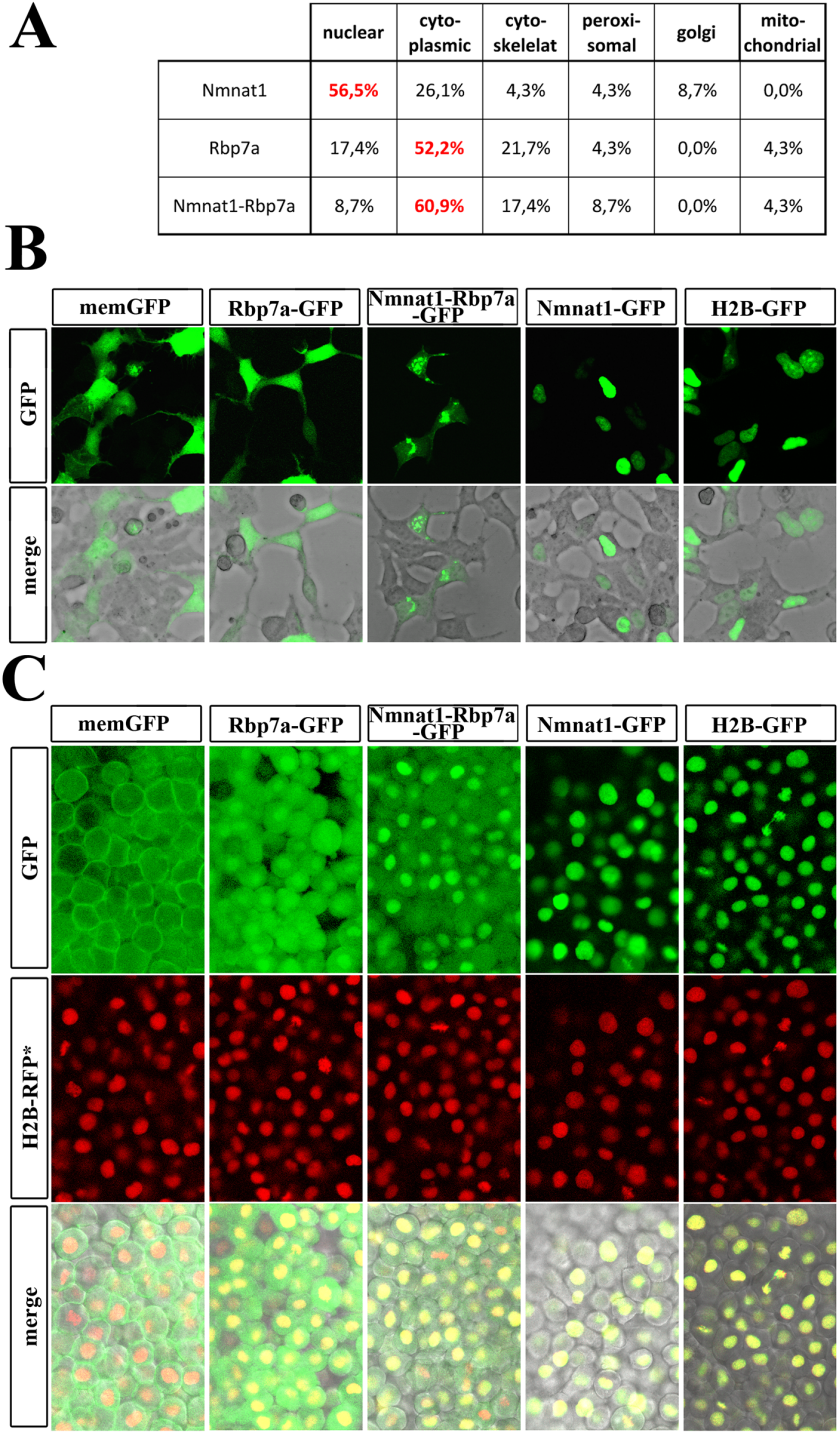
Subcellular localization of Nmnat1, Rbp7a and Nmnat1-Rbp7a proteins. [A] PSORT based predictions for sub-cellular location of Nmnat1, Rbp7a and Nmnat1-Rbp7a fusion. [B] Confocal image scans of HEK cells transfected with indicated fusion proteins. Note the strictly cytoplasmic and nuclear GFP-signals for Rbp7a-GFP and Nmnat1-Rbp7a-GFP, respectively. Nmnat1-Rbp7a-GFP transfected cells show weak GFP signals throughout the cytoplasm and stronger focal signals in association with the nuclei and cellular protrusions. [C] Confocal image scans of 6hpf embryo injected with mRNA encoding indicated GFP-tagged proteins. Co-injected mRNA encoding H2B-RFP (nuclear RFP, middle column) was used as loading control and to outline nuclei. Control injections of H2B-GFP and memGFP (membrane GFP) were used to document GFP/RFP co-expression. Rbp7a-GFP and Nmnat1-Rbp7a-GFP both showed nuclear and cytoplasmic localizations; but Nmnat1-GFP was only found in the nucleus.

**Fig 6:**
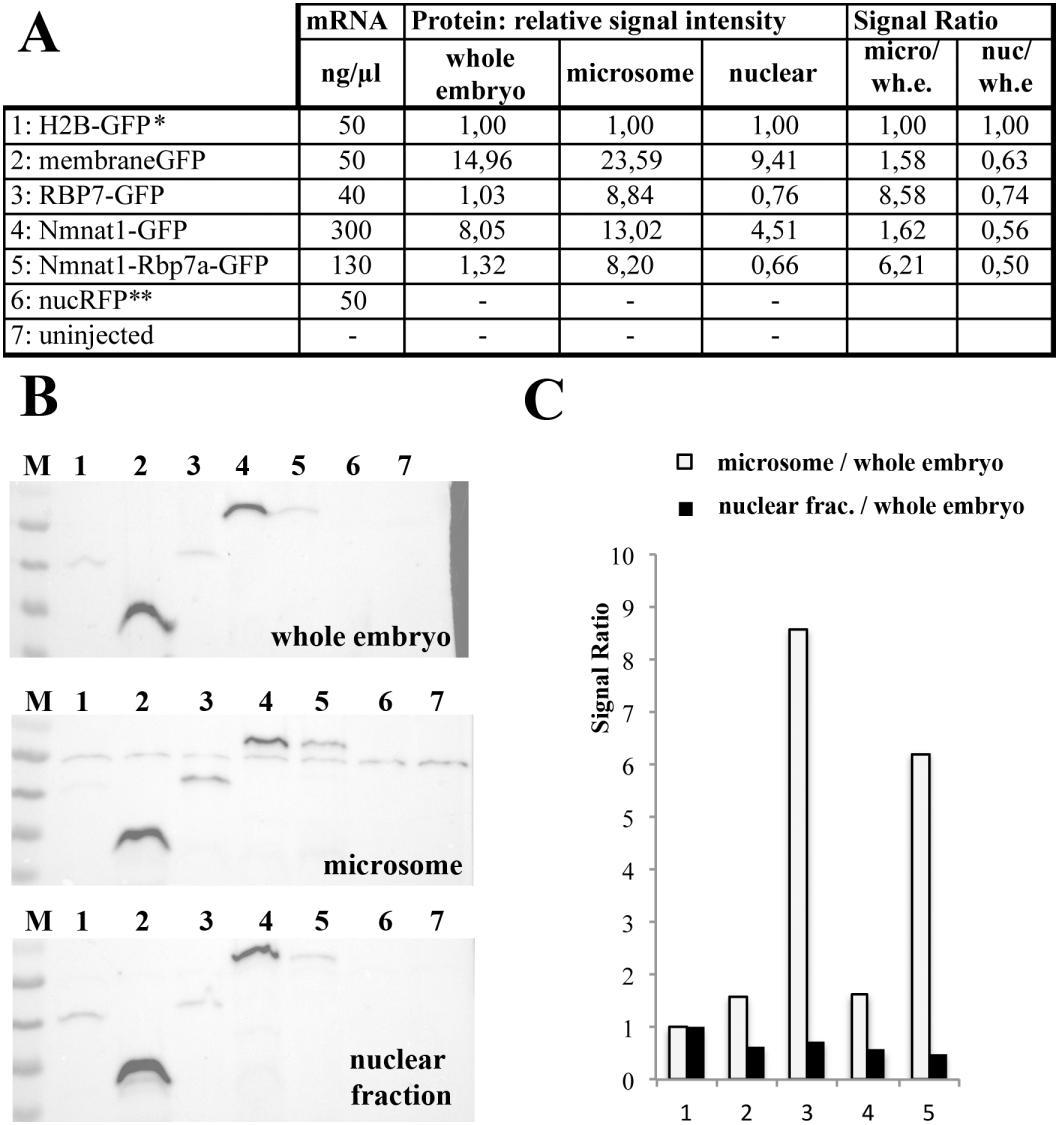
Rbp7a-GFP and Nmnat1-Rbp7a-GFP are enriched in microsome preparation. [A] Experimental overview indicating the encoded proteins, concentrations of injected mRNA (2nl/embryo) and data quantification of Western-Blot shown in [B]. Relative signal intensities always relate to H2B-GFP signals (*). Signal ratios correspond to the indicated quotient of t relative signal intensities. All mRNA were co-injected with 50ng/μl *nucRFP* mRNA (**). [B] Anti-GFP based Western-Blot analyses of whole embryo extracts (~1,7 embryos/lane), microsome preparation (~7,5 embryos/lane) and nuclear extracts (~1,5 embryos/lane). [C] Diagram showing indicated relative signal ratios shown in [A].

The mild increase in overall NAD^+^ levels seen in *nmnat1-rbp7a* injected embryos is unlikely to have a major impact on retinoid metabolism. However, there is evidence for organelle specific, non-redundant activities of Nmnat proteins [42–45]. In humans, the three NMNAT proteins have been shown to exert slightly different enzymatic activities at different subcellular localization [42, 43]. In particular, the use of tagged proteins revealed organelle specific targeting of Nmnat1 to the nucleus, of Nmnat2 to the Golgi complex and of Nmnat3 to the mitochondria. Therefore, we hypothesized that Nmnat1 and Nmnat1-Rbp7a also might differ in their sub-cellular localizations. This notion was supported by protein sequence based prediction analysis (PSORT server) that suggested a nuclear bias for Nmnat1 (56,5%) and a dominant cytoplasmic bias for Nmnat1-Rbp7a (60,9%) and Rbp7a (52,2%, Fig 5A). To verify these predictions, fusion constructs of full-length proteins tagged with C-terminal eGFP were generated and expressed in HEK cells (Fig 5B) as well as in zebrafish embryos (Fig 5C). For the zebrafish experiments, embryos at the 1–2 cell stage were injected with a mixture of two mRNAs, one encoding the GFP-tagged protein and the second encoding a nuclear specific RFP-variant (H2B-RFP) as an injection control. As described for the human fusion protein, zebrafish Nmnat1-GFP was strictly nuclear in both systems (Fig 5B and 5C compare with H2B-GFP). In contrast, Rbp7a-GFP showed the predicted cytoplasmic distribution in HEK cells and an additional slight nuclear enrichment in the early zebrafish embryo. Importantly, also the Nmnat1-Rbp7a-GFP fusion isoform was detected in both the nucleus and the cytoplasm. The data suggest an Rbp7a-dependend expansion of the NAD^+^ catalyzing activity into the cytoplasm. As the majority of cellular retinal-generating capacity resides in the ER, we speculated that Nmnat1-Rbp7a might function as a ER-specific NAD^+^ source [8]. To test this option, different cellular fractions prepared from embryos injected with mRNA encoding the different GFP-tagged proteins were analyzed by Western-blots using an GFP-antibody (summarized in Fig 6A, for details see Material and Methods). Importantly, signals for Rbp7a-GFP (lane 3) and Nmnat1-Rbp7a-GFP (lane 5) as compared to Nmnat (lane 4) were much stronger in the ER-containing microsome fractions as compared to whole embryo extracts and the nuclear/debris fractions (termed ‘nuclear fraction’, Fig 6B). Signal quantification revealed that in whole embryo extract the protein levels for Rbp7a-GFP and Nmnat1-Rbp7a-GFP were similar to that of H2B-GFP and roughly 6–8 times lower than those of Nmnat1-GFP (Fig 6A and 6B). In contrast, in the microsome preparation Rbp7a-GFP and Nmnat1-Rbp7a-GFP level were more than 8 times higher than that of H2B-GFP and only 1,5–1,6 lower than that of Nmnat1-GFP. The data correspond to a microsome specific enrichment of Rbp7a-GFP and Nmnat1-Rbp7a-GFP over H2B-GFP by factors of 8,6 and 6,2, respectively (Fig 6A and 6C). This suggests a similar association of Rbp7a and Nmnat1-Rbp7a with microsomal structures, presumably with the ER.

## Discussion

### Role of *rbp7a* in embryonic development

Retnoids play pivotal roles in vertebrate development. The hydrophobic nature of these compounds limits their solubility and diffusion in cellular environments. Thus animals have evolved specific binding proteins for these compounds. RBPs also control the access of specific retinoids to certain enzymes for esterification and redox reactions [12, 18, 46]. In particular, Rbp7 was shown to facilitate conversion of ROL to RE which is catalyzed by Lrat [17]. In mouse, *Rbp7* and *Lrat* mutants develop normally but show alter retinoid levels in adult liver and lactating mammary gland, which are major organs for retinoid storage and distribution [17, 47, 48]. Mammals are unique with respect to the placental maternal supply of retinoids. In oviparous animals, including the majority of vertebrates, the yolk is the only source for these compounds. This situation requires mechanisms that regulate and limit release of yolk-stored retinoids and conversion of these retinoids into RA. In Lratb-deficient zebrafish embryos the inability to convert free ROL into RE causes excess production of RA and as a consequence impairs development of the embryos [33]. Since Lrat and Rbp7 may act together to oppose Aldh1a1-dependent RA production, we speculated that loss of rbp7 function would lead to comparable phenotypes. However, unlike the Lratb deficient zebrafish embryos in which retinoids are shifted towards increased RA production, our analyses revealed no evidence for changed embryonic retinoid levels after *rbp7a* knockdown. A possible explanation for the discrepancy between the *lratb* and *rbp7a* deficient phenotypes is that esterification of ROL still takes place in the absence of Rbp7 proteins, as has been reported for mammals [17, 47, 48]. Additionally, the specific regulation of these genes may contribute to this difference. The much broader embryonic expression of *lratb* as compared to *rbp7a* suggests Lratb interaction with additional early-regulated Rbps, in particular with Rbp5, Rbp7b and Rbp4 [20, 22, 23, 33]. Thus, *lratb* knockdown results in an overall change of retinoid homeostasis in all embryonic compartments that is detectable by the applied HPLC method. However, such whole-embryo HPLC measurements cannot assess biologically relevant changes in retinoid levels in defined embryonic compartments. In contrast to *lratb*, expression of *rbp7a* was mainly found in the YSL and in forerunner cells during blastula and gastrula stages, and no specific signals could be detected in wild type embryos older than 12 hpf [23]. Thus, the loss of *rbp7a* is not necessarily expected to affect overall retinoid composition of the embryo. In line with the concept of spatially and temporally confined Rbp7a activities, the strong *rbp7a* signals in the forerunner cells have recently been associated with a local role during establishment of left-right asymmetry [24]. Zebrafish forerunner cells form motile cilia whose activity is required to break left-right symmetry. In particular it was found that shortening of these cilia in rbp7a morphants correlated with a loss of unilateral activation of the Nodal ligand *spaw* and reversed heart looping. While heart-looping phenotypes were not specifically analyzed in this work, we also found reversed or medial hearts in more than 40% of embryos in which high *rbp7a*-morpholino amounts were co-injected with a morpholino targeting p53. Interestingly, all MO-Δex2/MO-p53 (n>100) co-injected embryos at 4dpf developed severe pericardial and abdominal edema while the majority of MO-ATG/MO-p53 injected embryos had a normal appearance (not shown). These data hint for a potential late embryonic Nmnat1-Rbp7 requirement and they suggest rbp7a-exon2 as a promising target for addressing these functions by genetic rather than knock-down approaches.

### Role of *rbp7a* in Nodal/FoxH1 modulated RA production

Here we show that *rbp7a* transcript levels were increased in Nodal/FoxH1 mutants and that this increase was not associated with a major change in the pattern of rbp7a mRNA localization. Currently neither the importance of the *rbp7a* regulation nor the underlying mechanism is clear. In our studies, we identified a FoxH1 consensus sequence close to the transcriptional start site of *rbp7a* that was recognized by FoxH1 in EMSA studies and that in ChIP analyses showed *in vivo* occupancy by GFP-tagged FoxH1. These data are consistent with a potentially direct repression of *rbp7a* by Foxh1. Foxh1 is supposed to act mainly as a transcriptional activator [25, 49, 50] and very few examples for a direct FoxH1-dependent repression have been described so far. In mouse, FoxH1 was shown to repress *mix1l* by recruiting the transcriptional repressor Gsc to a FoxH1 regulated enhancer element [51]. In zebrafish, FoxH1 was found to repress endothelial expression of *flk1*, encoding the major receptor for VEGF [52]. More recently, a genome wide analyses of Foxh1 targets in *Xenopus* provided evidence for Foxh1 activities in direct repression and activation of target genes that appear to be independent of Nodal-signaling [53]. As *foxH1* is broadly expressed in the early embryo, corresponding Foxh1 targets in *foxh1* mutants are expected to display a change in the expression levels but not necessarily a changed expression pattern. While previous studies also noted impact of Nodal signaling on the regulation of ubiquitously expressed gene, the underlying mechanisms have not been addressed to far [54]. Our results suggest the *rbp7a* genes as a promising candidate for addressing the molecular mechanism of Nodal/FoxH1 dependent gene repression.

What could be the function of the repression of *rbp7a* by Nodal signaling. Previous studies in mice revealed a crosstalk between Nodal and RA signaling during orofacial development [37]. In particular, it was shown that the enzyme Aldh1A2, which converts retinal to RA, was a direct target gene of the Nodal pathway and that this activation was mediated by the transcription factor FoxH1 [37]. Consistent with a similar cross talk between Nodal and RA-signaling in early zebrafish development we found that *aldh1A2* expression is reduced in Nodal-signaling deficient MZ*oep* mutants and in MZ*sur/foxH1* mutants during gastrulation. An attractive model for explaining these complementary Nodal-activities on *rbp7a* and *aldh1A2* expression is that Nodal synchronizes two competing pathways impacting on RA production. While induction of Aldh1A2 by Nodal/Foxh1 will directly increase RA catalysis, the simultaneous block of Rbp7a might indirectly support RA synthesis by reducing Lrat catalyzed removal of free ROL.

### Nmnat1-Rbp7 is an evolutionarily conserved protein

In this study we described a conserved chimeric transcript encoding a fusion of Nmnat1 and Rbp7 proteins. Our cDNA sequencing data from zebrafish, chick and mouse isoforms suggested that *nmnat1* and *nmnat1-rbp7* mRNAs are formed from the same pre-RNA by alternative splicing and that the *rbp7* transcript is generated from a separate promoter. The different time windows of embryonic expression and tissue specific differences in the adult expression demonstrated an independent regulation of zebrafish *nmnat1*, *nmnat1-rbp7a* and *rbp7a* transcription and splicing.

What kind of adaptive evolution had occurred in forming the chimeric gene *nmnat1-rbp7* and had conserved it in different species? Our analyses of the Nmnat1-Rbp7a fusion protein showed similar activities as for Nmnat1 in NAD^+^ synthesis. In addition, our studies with GFP-tagged proteins suggest different subcellular localization of these proteins. The *in vivo* localization analyses revealed that the mature Nmnat1 protein was restricted to the nucleus and that Nmnat1-Rbp7a and Rbp7a were localized in the cytoplasm and in the nucleus. Furthermore, our Western-blot analyses showed enrichment of Nmnat1-Rbp7a and Rbp7a in microsome preparations. Functional relevance of the distinct subcellular localization of NAD^+^ catalyzing enzymes is currently not clear. Studies that revealed differences in the catalytic activities of NMNAT proteins argued against a redundant contribution to cellular NAD^+^ homeostasis and rather suggested organelle specific activities [42–45]. The localization of Nmnat1-Rbp7a complements the previously reported localization of human NMNAT proteins in the nucleus, Golgi and mitochondria. Considering the *nmnat1* independent regulation of *nmnat1-rbp7* expression, our data support the previously suggested model of organelle-specific NMNAT functions. Notably, the majority of cellular retinal-generating capacity resides in microsomes and Rbp1 has been shown to interact with several microsome-associated proteins involved in retinoid metabolism [8, 55]. The results support direct Rbp7 and Nmnat1-Rbp7 interactions with microsome associated Lrat and short-chain dehydrogenase/reductases (SDRs) and thereby suggest chaperone functions in RE or retinal catalysis, respectively. In this case, Nmnat1-Rbp7a protein would serve as a microsome specific source for NAD^+^. As interaction between Rbp1 and SDR was found to be NAD^+^ dependent, a direct association between SDRs and Nmnat1-Rbp7 as a local NAD^+^ source might shift retinoid metabolism towards increased levels of retinal and RA.

Our analyses revealed expression of *nmnat1-rbp7a* in the adult eye and liver. Remarkably, several recent studies have shown that, in humans, mutations in *NMNAT1* result in lower levels of NAD^+^ and have been associated with Leber congenital amaurosis, a severe form of inherited blindness [56–59]. This disease is associated with pigmentary changes, attenuated retinal blood vessels and optic disc pallor. Considering the similarity of NAD^+^ catalysis activity between Nmnat1 and Nmnat1-Rbp7a fusion, and the cytoplasmic localization of Nmnat1-Rbp7a-GFP, these studies hint at a potential role of the fusion isoform protein in retinoid processing.

## Supporting Information

S1 Fig Loss of *rbp7a*-exon2 in MO*-Δex2* injected embryos. Reverse transcriptase PCR with indicated primers spanning *rbp7a* exon 2. The cDNA was prepared from the stage 6hpf MO injection and wild type embryos. The primers amplify the region between the exon1 (Pr1) and exon3 (Pr3).
(DOCX)

S2 Fig HPLC profiles of embryonic retinoid and NAD^+^ measurements. [A] HPLC analysis of retinoids in lipid extracts of 14hpf zebrafish embryos. Polar and non-polar retinoids were extracted from 100 embryos as previously described [33]. Lipid extracts were suspended in 200 μl running solvent (hexane/acetylacetate 71/19 (v/v) with 7.5 μl acetic acid per 100 ml). 100 μl were injected into the HPLC system and developed with running agent with isocratic flow of 1.4 ml/min. [A] Spectral characteristics of retinyl esters (peak 1), all-trans-retinol (peak 2) and all-trans-retinoic acid (peak 3). [B] A representative HPLC chromatogram at 360 nm is shown. The inset gives a magnification of the HPLC trace from min 8 to 8.5. For quantification of the amounts of different retinoids, the HPLC system was scaled with authentic standard substances (purchased from Sigma). Quantification was performed as based on three independent samples (n = 100 embryos). [C] HPLC analysis of NAD^+^ obtained from 6hpf embryos (n = 100). The NAD^+^ extraction protocol was described previously [34]. A representative HPLC chromatogram at 259 nm is shown. Runs were performed isocratic with a flow rate of 1 ml/min for 6 minutes using a 0.05 M phosphate buffer pH 7.0 containing 3% acetonitrile.
(DOCX)

S1 Table PCR-Primers used for RT-PCR and qPCR analyses. (DOCX)

## Abbreviations

ADH: alcohol dehydrogenase
ALDH: Aldehyde-dehydrogenase
ChIP: Chromatin Immunoprecipitation
gsc: goosecoid
FoxH1: forkhead box factor H1
hpf: hours post-fertilization
HPLC: High-performance liquid chromatography
IRBP: Interphoto-receptor retinoid binding protein
LRAT: lecithin-retinol acyltransferase
NAD^+^: Nicotinamide adenine dinucleotide
NMNAT1: nicotinamide mononucleotide adenylyltransferase 1
MZ: maternal zygotic
RA: all-*trans*-retinoic acid
RARs: retinoic acid receptors
RBP: Retinoid binding protein
RDH: retinol dehydrogenase
RE: retinyl ester
ROL: all-trans-retinol
RXRs: retinoid X receptors
SDR: short-chain dehydrogenase/reductase
Shh: Sonic hedgehog
YSL: yolk syncytial layer
