# Supplementary material for: Nmnat1-Rbp7 Is a Conserved Fusion-Protein That Combines NAD+ Catalysis of Nmnat1 with Subcellular Localization of Rbp7
Source: PLoS One. 2015 Nov 30;10(11):e0143825. doi: 10.1371/journal.pone.0143825 (PMC4664474; doi:10.1371/journal.pone.0143825)
Supplement: S1 Fig — Reverse transcriptase PCR with indicated primers spanning rbp7a exon 2. The cDNA was prepared from the stage 6hpf MO injection and wild type embryos. The primers amplify the region between the exon1 (Pr1) and exon3 (Pr3). (DOCX) [file pone.0143825.s001.docx]

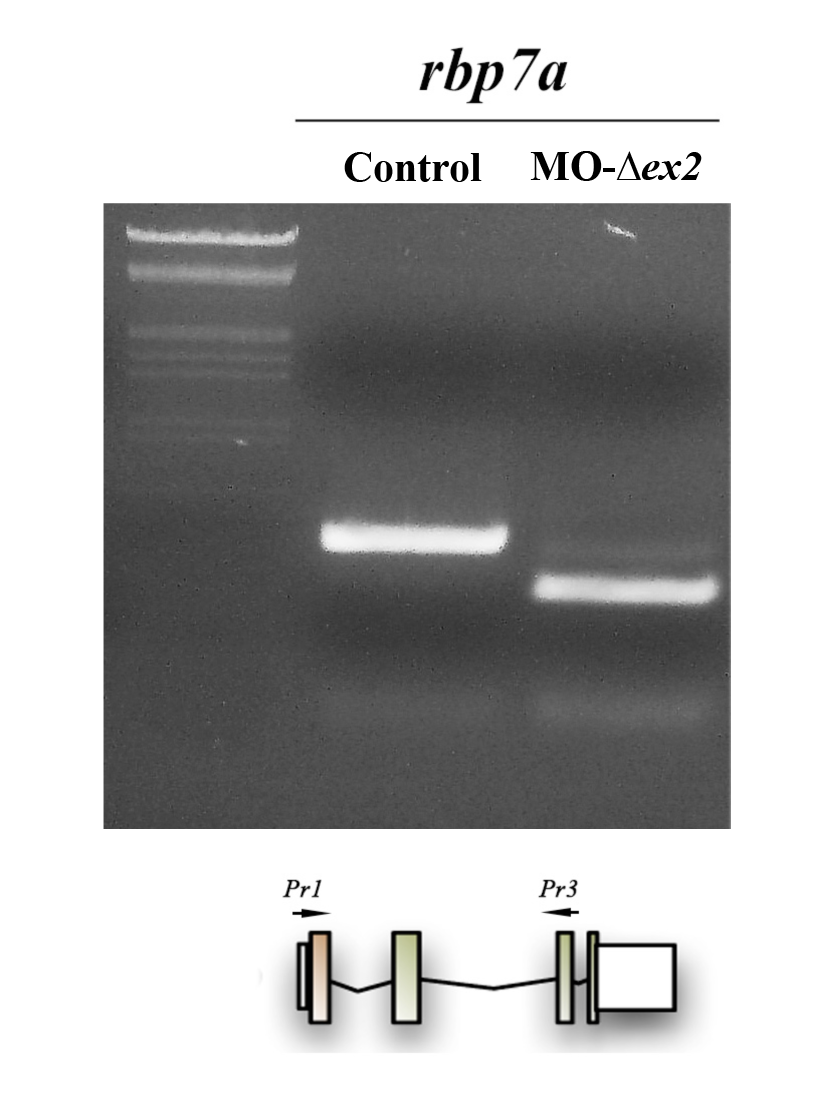


**S1 Fig. Loss of *rbp7a*-exon2 in MO*-∆ex2* injected embryos**

Reverse transcriptase PCR with indicated primers spanning *rbp7a* exon 2*.* The cDNA was prepared from the stage 6hpf MO injection and wild type embryos. The primers amplify the region between the exon1 (Pr1) and exon3 (Pr3).
