# Supplementary material for: Nmnat1-Rbp7 Is a Conserved Fusion-Protein That Combines NAD+ Catalysis of Nmnat1 with Subcellular Localization of Rbp7
Source: PLoS One. 2015 Nov 30;10(11):e0143825. doi: 10.1371/journal.pone.0143825 (PMC4664474; doi:10.1371/journal.pone.0143825)
Supplement: S2 Fig — [A] HPLC analysis of retinoids in lipid extracts of 14hpf zebrafish embryos. Polar and non-polar retinoids were extracted from 100 embryos as previously described [33]. Lipid extracts were suspended in 200 μl running solvent (hexane/acetylacetate 71/19 (v/v) with 7.5 μl acetic acid per 100 ml). 100 μl were injected into the HPLC system and developed with running agent with isocratic flow of 1.4 ml/min. [A] Spectral characteristics of retinyl esters (peak 1), all-trans-retinol (peak 2) and all-trans-retinoic acid (peak 3). [B] A representative HPLC chromatogram at 360 nm is shown. The inset gives a magnification of the HPLC trace from min 8 to 8.5. For quantification of the amounts of different retinoids, the HPLC system was scaled with authentic standard substances (purchased from Sigma). Quantification was performed as based on three independent samples (n = 100 embryos). [C] HPLC analysis of NAD+ obtained from 6hpf embryos (n = 100). The NAD+ extraction protocol was described previously [34]. A representative HPLC chromatogram at 259 nm is shown. Runs were performed isocratic with a flow rate of 1 ml/min for 6 minutes using a 0.05 M phosphate buffer pH 7.0 containing 3% acetonitrile. (DOCX) [file pone.0143825.s002.docx]

**
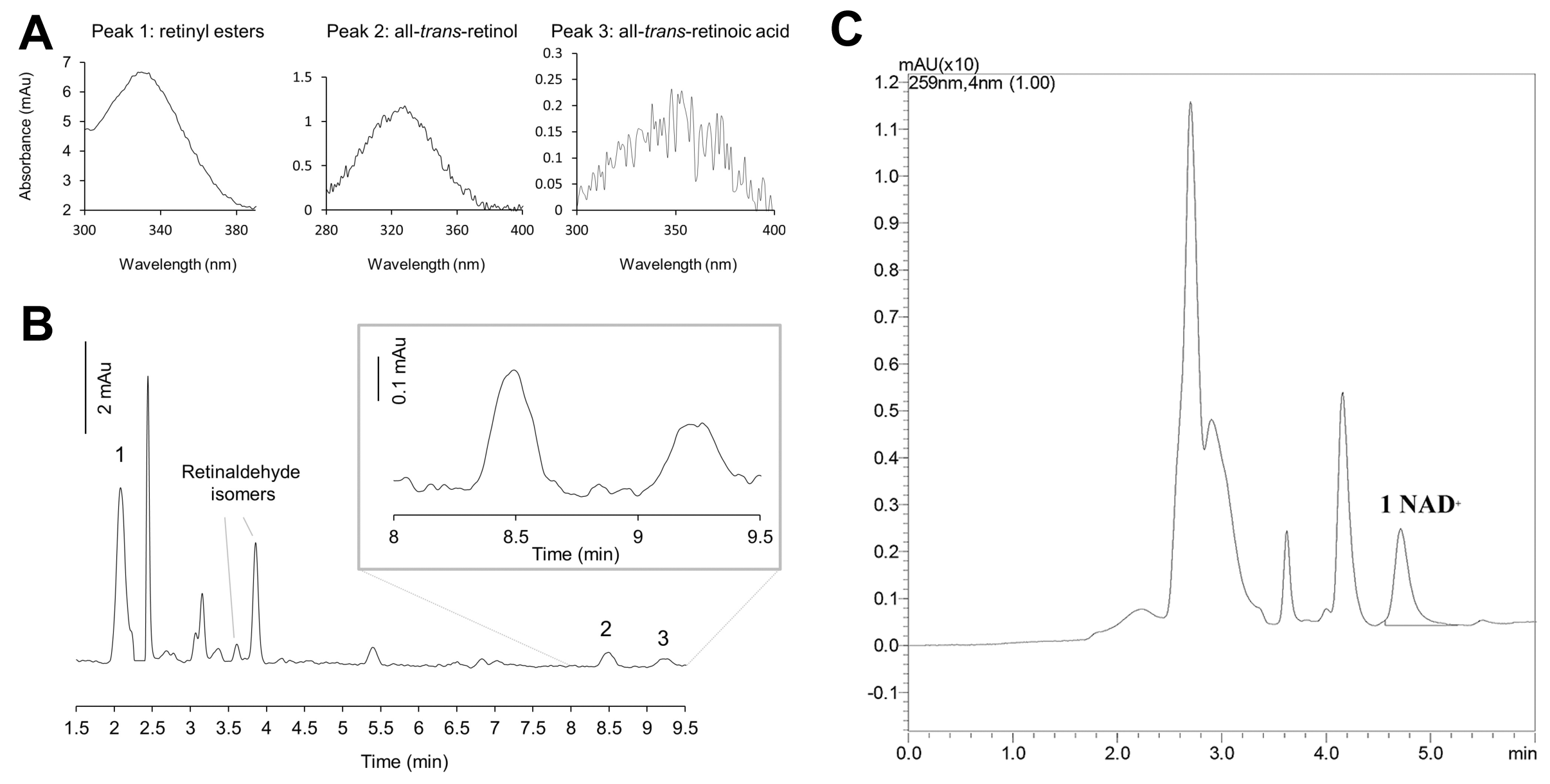
**

**S2 Fig. HPLC profiles of embryonic retinoid and NAD^+^ measurements.**

[A] HPLC analysis of retinoids in lipid extracts of 14hpf zebrafish embryos. Polar and non-polar retinoids were extracted from 100 embryos as previously described [33]. Lipid extracts were suspended in 200 μl running solvent (hexane/acetylacetate 71/19 (v/v) with 7.5 μl acetic acid per 100 ml). 100 μl were injected into the HPLC system and developed with running agent with isocratic flow of 1.4 ml/min. [A] Spectral characteristics of retinyl esters (peak 1), all-trans-retinol (peak 2) and all-trans-retinoic acid (peak 3). [B] A representative HPLC chromatogram at 360 nm is shown. The inset gives a magnification of the HPLC trace from min 8 to 8.5. For quantification of the amounts of different retinoids, the HPLC system was scaled with authentic standard substances (purchased from Sigma). Quantification was performed as based on three independent samples (n=100 embryos). [C] HPLC analysis of NAD^+^ obtained from 6hpf embryos (n=100). The NAD^+^ extraction protocol was described previously [34]. A representative HPLC chromatogram at 259 nm is shown. Runs were performed isocratic with a flow rate of 1 ml/min for 6 minutes using a 0.05 M phosphate buffer pH 7.0 containing 3% acetonitrile.
