# Supplementary material for: Nmnat1-Rbp7 Is a Conserved Fusion-Protein That Combines NAD+ Catalysis of Nmnat1 with Subcellular Localization of Rbp7
Source: PLoS One. 2015 Nov 30;10(11):e0143825. doi: 10.1371/journal.pone.0143825 (PMC4664474; doi:10.1371/journal.pone.0143825)
Supplement: S1 Table — (DOCX) [file pone.0143825.s003.docx]

**S1 Table. PCR-Primers used for RT-PCR and qPCR analyses**

| **Primers** | **Forward** | **Reverse** |
| --- | --- | --- |
| **Full length cloning** | | |
| *zf-rbp7a* | ATTGTTTGCACTTTGTTTT | TTATGCACATAATAGGCTTT |
| *zf-nmnat1* | TCAGGTTTTCTGTTTCTTTACTA | GAAGCAGCTTTACTCAGTGTTG |
| *zf-nmnat1-rbp7a* | TTGCAGCTGTCCTGAGAAG | AACAACATGTATATGTAGGCATCA |
| *ch-nmnat1-rbp7* | GGGAGGAGACTGCAGGGACG | AAGATGTTCTGTGGTGTTTATTAGCA |
| *m-nmnat1-rbp7* | GGCCAATGAGAGCAAGGCGG | ACCACTTGAGCAGGTTTATTGGAGC |
| **Semi quantitative RT-PCR** | | |
| *Pn2, zf-nmnat1 ex2* | TCGCTTGGAGATGGCTAGAC |  |
| *Pn5, zf-nmnat1 ex5* |  | ACTGATGTTCTTTCGGTGTT |
| *Pr1, zf-rbp7a ex1* | TTGCACTTTGTTTTCCACTGG |  |
| *Pr3, zf-rbp7a ex3* |  | CATCCTCAATCCAGTGTGCC |
| *mn4, m-nmnat1 ex4* | GAAAAGCCTGGGCGGAAGAG |  |
| *mn5, m-nmnat1 ex5* |  | GCCAGCCCGAGTGATACAGATG |
| *mr1, m-rbp7 ex1* | TGCGGACAGACACCAAACCA |  |
| *mr2, m-rbp7 ex2* |  | AGGTAGTTCCTGAGGCTGCTGC |
| *cr1,ch-rbp7 ex1* | CTGATTCTTAATTTCTGCTTTTTG |  |
| *cr2, ch-rbp7 ex3* |  | TGTGCTTGTGGTATGGATGGAA |
| *cf5, ch-nmnat1 ex4* | TGGAAAAAATGTCCCACAGGTT |  |
| *cn5, ch-nmnat1 ex5* |  | CCCGAACCACATCAGGCACTA |
| **ChIP-qPCR** | | |
| *Zf-rhodopsin* | GACTCCACACAATCTGCAACAT | ACCACCTACGCTAAAGAAACCA |
| *rbp7a site 1* | TAGTATTAACTAATGCAGCATAACT | AGGTTCTGCTTAGCTTGTGTAT |
| *rbp7a site 2* | ATTCCCCAGTGTTTCCCCTAC | TAAAAGGTTTGAGCTGTAAACATAG |
| *rbp7a site 3* | TTATGACAGCCAGTTGTAATAAG | AGCTGGGCTAAGTTTGGTCTT |
| *rbp7a site 4* | TTAACATGCATTCCTAATTGGT | TGAGATACTGATGAGTGTTGTGTA |
| *PD_gsc* | TATAATCAACCGTGCCATGC | ACCATTAGCGGCCCTATTCT |
| *PP_gsc* | AGCTATTGTGAACCAAATAATGTGA | GGCTGGACTTAGGGTTAGCA |
| *lhx1a* | TCAATGCTTTGCCAGGGTAGTT | CCACTGCAAACACACTCGGCGT |
| **RT-qPCR** | | |
| *beta-actin (ref1)* | CTGCTCTGTATGGCGCATTGAC | GTTAGACAACTACCTCCCTTTGCC |
| *ef1a (ref2)* | TCTCTACCTACCCTCCTCTTGGTC | TTGGTCTTGGCAGCCTTCTGTG |
| *rpb7a* | *Pr1, zf-rbp7a ex1* | *rbp7a-ex2*: TAGCTTTAGGGCGATCTTGC |
| *nmnat1-rbp7a* | *Pn2, zf-nmnat1 ex2* | *rbp7a-ex2*: TAGCTTTAGGGCGATCTTGC |
| *nmnat1* | TGTGCTGGAGTCGTTTGG | *Pn5, zf-nmnat1 ex5* |
| *aldh1a2* | ATGTCCTGCCAGTGTCCTTTCG | TCAGTCCGATCTCTCCGAGTTCAC |
